# Alzheimer's Disease: A short introduction to the calmodulin hypothesis

**DOI:** 10.3934/Neuroscience.2019.4.231

**Published:** 2019-10-12

**Authors:** Danton H. O'Day

**Affiliations:** 1 Cell and Systems Biology, University of Toronto, Toronto, Ontario, Canada M5S 3G5; 2 Department of Biology, University of Toronto Mississauga, Mississauga, Ontario, Canada L5L 1C6

**Keywords:** Alzheimer's disease, calmodulin hypothesis, calmodulin-binding, amyloid beta, neurofibrillary tangles, CaMKII, calcineurin, risk factor proteins, hallmarks

## Abstract

At the cellular level, Alzheimer's disease (AD) is characterized by the presence of intracellular plaques containing amyloid beta (Aβ) protein and neurofibrillary tangles consisting of phospho-tau (p-tau). These biomarkers are considered to contribute, at least in part, to the neurodegenerative events of the disease. But the accumulation of plaques and tangles is widely considered to be a later event with other factors likely being the cause of the disease. Calcium dysregulation—the unregulated accumulation of calcium ions—in neurons is an early event that underlies neurodegeneration. In 2002, O'Day and Myre extended this “Calcium Hypothesis” to include calmodulin (CaM) the primary target of calcium, suggesting the “Calmodulin Hypothesis” as an updated alternative. Here we overview the central role of CaM in the formation of the classic hallmarks of AD: plaques and tangles. Then some insight into CaM's binding to various risk factor proteins is given followed by a short summary of specific receptors and channels linked to the disease that are CaM binding proteins. Overall, this review emphasizes the diversity of Alzheimer's-linked CaM-binding proteins validating the hypothesis that CaM operates critically at all stages of the disease and stands out as a potential primary target for future research.

## 1. Introduction

As the primary cause of dementia, Alzheimer's disease (AD) affects approximately 50 million people worldwide, a number that is projected to increase dramatically due to the age-dependency of this disease [1]. Research into the primary hallmarks of the disease—amyloid beta plaques and phospho-tau neurofibrillary tangles—has failed to produce a therapeutic approach to slowing let alone stopping AD. GWAS research has identified numerous genes, and their corresponding proteins, that are risk factors for late onset AD, the most common form of the disease. Coupled with aging, lifestyle risk factors, including smoking, diet, exercise and others, have also been identified as significant contributors in the development of AD [1]. The problem lies in linking all of these elements together to establish a logical and accurate timeline for disease onset and progression—a long and challenging journey.

As researchers continue to find new factors implicated in the disease, one theme established years ago continues to be central: the dysregulation of calcium signaling. The “Calcium Hypothesis” purports that the unregulated influx of calcium ions into the cytoplasm of neurons is an early event that leads first to the production of amyloid beta followed by neurofibrillary tangle formation as underlying changes that drive neurodegeneration [2],[3]. In 2004, O'Day and Myre first presented arguments that calmodulin (CaM), the main calcium-binding protein and regulator of is function, is the primary target of the calcium dysregulation [4]. What's more, as continued research by others added strength to the “Calcium Hypothesis”, it became clear that many of the affected molecular events were regulated by CaM. After an overview of CaM-binding proteins involved in the production of the classic hallmarks of AD, the association of CaM with risk factor proteins as well as in the regulation of receptors, ion channels and other critical proteins is reviewed. The accumulating data continues to emphasize the central role of CaM as an early and central regulator of the early and late events of AD. Here, we overview the diversity of proteins that bind to CaM with the goal of promoting more research on the role of CaM in the Alzheimer's disease process.

## 2. From the calcium hypothesis to the calmodulin hypothesis

In 1994, Khachaturian first proposed the “Calcium Hypothesis” for AD: disruption of Ca^2+^ levels in brain cells leads to the neuronal deterioration of AD [2]. The central role of Ca^2+^ signaling in neurotransmitter synthesis and release, membrane excitability and energy metabolism has been exhaustively studied [5]. Ca^2+^ signaling is also central to learning and memory and, since the normal, intracellular concentration of this divalent cation is controlled in a range of 10^−7^ to 10^−8^ mol., even slight but persistent disruptions can be detrimental [6]. An in-depth re-evaluation of the “Calcium Hypothesis” stated, “Growing evidence now supports early presymptomatic roles for dysregulated cellular Ca^2+^ homeostasis in promoting amyloidogenesis, cytoskeletal pathologies, mitochondrial dysfunction, synaptic transmission and plasticity dysfunction, and oxidative stress.” [1]. While the updated version of the hypothesis revealed multiple ways dysregulated Ca^2+^ levels can play a role in neurodegeneration, it failed to recognize the depth of the roles CaM plays in Ca^2+^ signaling.

Ca^2+^ does not work alone but through its binding to targets, predominantly Ca^2+^-binding proteins. CaM is arguably the predominant and primary Ca^2+^-binding protein in all eukaryotes and is a critical Ca^2+^ sensor and effector in the brain where it binds to essential proteins involved in synaptic functions, learning and memory [7]. As one example, alterations in Ca^2+^ signaling can lead to memory loss through the augmentation of LTD caused by the activation of calcineurin (CaN; PP2B), the sole CaM-dependent phosphatase [6].

## 3. Calmodulin works by regulating cam-binding proteins

**Figure 1.**
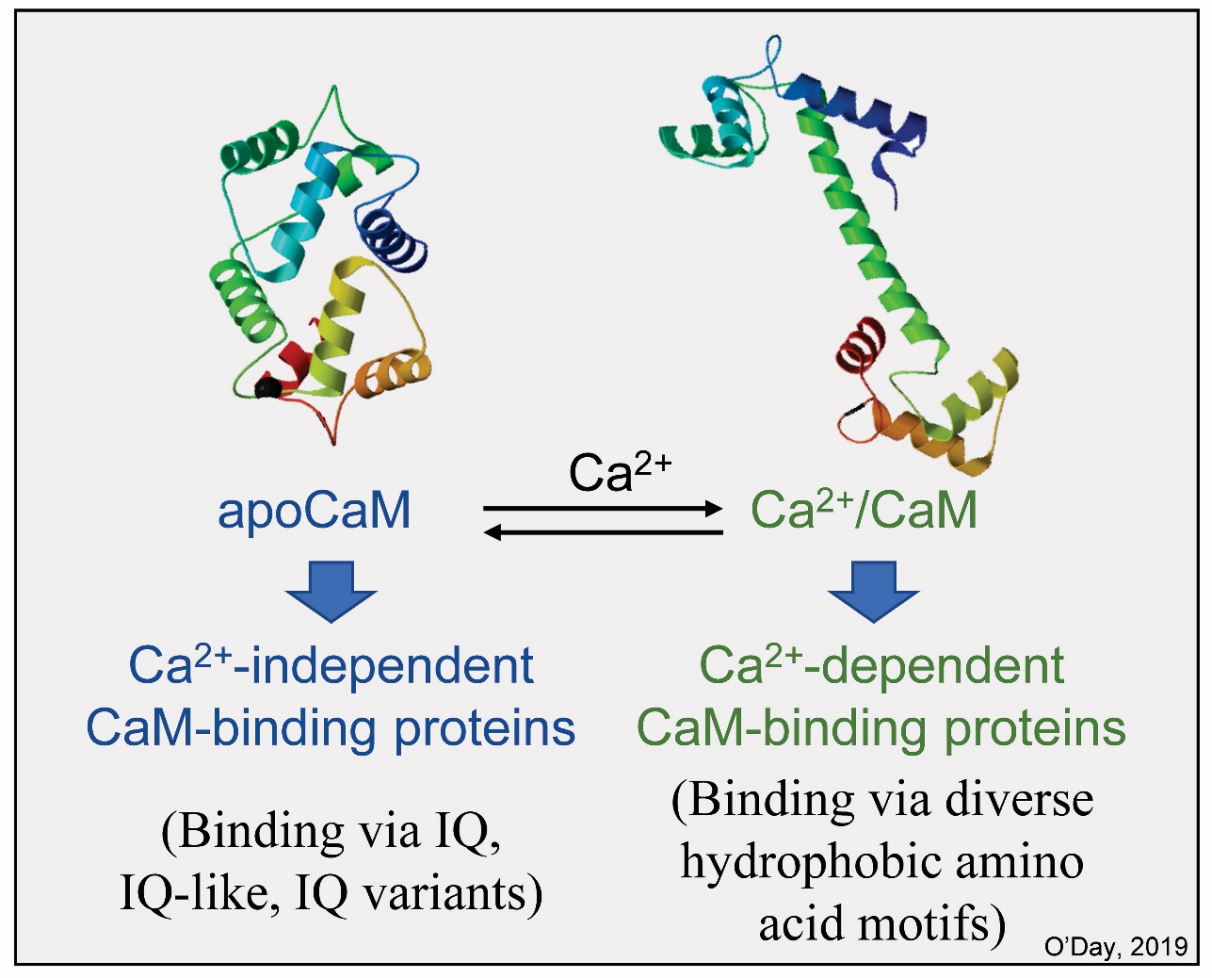
Apo-calmodulin and calcium-bound calmodulin use different motifs to bind target proteins.

CaM is a comparatively small, 152 amino acid Ca^2+^-binding protein that can bind to its target CaM-binding proteins (CaMBPs) either in a Ca^2+^-free (apo-CaM) state or when it is bound to Ca^2+^ (Ca^2+^/CaM). The way Ca^2+^ regulates CaM and the diverse ways it binds to its CaMBPs has been extensively reviewed [8]–[11]. Binding to Ca^2+^ leads to a dramatic conformational change in CaM, transforming its ability from the Ca^2+^-free binding of a select group of proteins to the Ca^2+^-dependent binding of a much larger set of proteins (Figure 1). In total, CaM can bind to well over 300 different proteins, many of which are localized in the brain. It is important to note that rather than binding via a specific recognition sequence as found in other regulatory proteins, CaM, especially Ca^2+^-bound CaM, instead recognizes variable motifs that share some common attributes rather than precisely defined sequences [10],[11]. These binding motifs differ significantly for Ca^2+^-independent versus Ca^2+^-dependent binding [8],[9].

Ca^2+^-free binding to apo-CaM involves IQ ([FILV]Qxxx[RK]Gxxx[RK]xx[FILVWY]), IQ-like ([FILV]Qxxx[RK]Gxxxxxxxx) motifs or the less-well-understood IQ variants. Ca^2+^-dependent binding is primarily dependent on the arrangement of hydrophobic residues of which multiple canonical motifs are known based on the positions of those amino acids (e.g., 1–10, 1–5–10, 1–12, 1–8–14, etc.) while new non-canonical motifs are being discovered regularly. Various algorithms can be used to identify putative or potential CaM-binding domains (CaMBDs) but, as with any domain, final verification requires the analysis of deletion constructs and other molecular approaches. For the majority of cases, while the binding domain may not have been experimentally verified, actual CaM-binding is validated by the direct binding of the CaMBP to CaM in the presence or absence of Ca^2+^ ions.

## 4. Calmodulin and the hallmarks of AD

**Figure 2.**
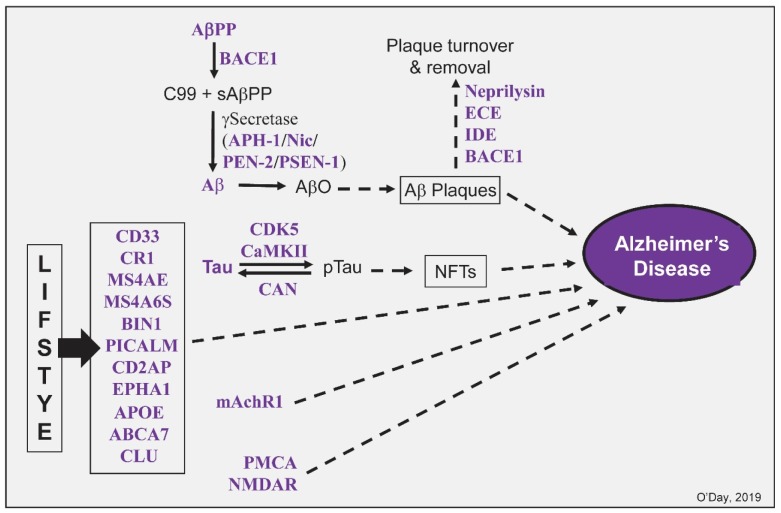
Some calmodulin binding proteins linked to events in Alzheimer's disease. Putative and proven calmodulin binding proteins are indicated in purple. See text for details.

Plaques and tangles— identified by Alois Alzheimer, the physician who discovered and named the disease—are widely regarded as two central hallmarks of Alzheimer's disease [12]. There is extensive evidence that CaM functions at every step in the early stages of plaque formation. Extracellular plaques form by the two-stage hydrolysis of amyloid beta precursor protein (AβPP) to produce Aβ which oligomerizes then associates with other components. AβPP binds to CaM [13],[14] (Figure 2). Canobbio et al (2011) further revealed the treatment of human platelets with W7, a calmodulin inhibitor, promoted the non-amyloidogenic processing of APP providing an example where interference of CaM function may be a means of reducing plaque load [14]. β-secretase (BACE1) functions to produce a membrane bound C99 fragment and a released soluble fragment of AβPP (sAβPP). Research has shown that BACE1 not only binds to CaM, its activity is significantly increased by CaM in a dose-dependent manner [15].

After BACE1, γ−secretase, a heterotetrameric protein, cleaves Aβ fragments of various sizes and toxicities which then oligomerize. Aβ binds to CaM Ca^2+^-dependently [16]. More critically, CaM binds with high affinity to its neurotoxic domain (Aβ25-35) inhibiting Aβ polymerization. Currently, the formation of toxic Aβ oligomers (AβO) is considered to be a crucial step in plaque formation [17]. It should be noted that Aβ has other important roles in AD some of which, as discussed for PMCA below, involve CaM.

γ−secretase consists of four subunits: anterior pharynx-defective 1 (APH-1), nicastrin (Nic), presenilin (PSEN-1), and presenilin enhancer 2 (PEN-2). APH-1, Nic and PEN-2 all possess CaMBDs with canonical motifs [18]. PSEN-1 is an experimentally verified CaMBP [19]. The role of CaM in later stages of plaque formation remains to be studied but there is some insight into its function in plaque removal. Four enzymes linked to plaque or Aβ turnover have been identified that bind to CaM: neprilysin, endothelin-converting enzymes (ECE), insulin degrading enzymes (IDE) and BACE1 [18]. Neprilysin, ECE and IDE possess canonical CaMBDs but remain to be validated as functional CaMBPs [18].

The second hallmark of AD is the formation of intracellular neurofibrillary tangles (NFTs). The phosphorylation of tau displaces it from microtubules allowing the p-tau to polymerized into tangles in a multistep process. CaM is involved in various aspects of neurofibrillary tangle formation. Several studies have shown that tau binds to CaM in a Ca^2+^-dependent manner and this association prevents tau binding to microtubules [20],[21]. The phosphorylation of tau, a prelude to fibrillary tangle formation, involves at least two CaM-dependent kinases: Ca^2+^/CaM-dependent protein kinase II (CaMKII) and cyclin-dependent kinase 5 (CDK5) [18]. CDK5 binds CaM in a Ca^2+^-independent manner [22]. In addition, CaN is a well-established CaMBP that has been historically linked to tau dephosphorylation [23]. The contradictory effects of CaMKII and CaN are well established making them appealing targets for the disruption of p-tau formation. Furthermore, specific inhibitors of these two enzymes, as well as CDK5, exist offering a means to test their efficacy in preventing NTF formation and/or the progression of AD. The various roles of CaMKII and CaN in Alzheimer's disease have been reviewed [24],[25].

## 5. Calmodulin binding to risk factor proteins

Numerous Genome Wide Association Studies (GWAS) have identified and continue to identify genes and their proteins that are risk factors for late onset AD [26]. Of these, 11 have been identified as validated or potential CaMBPs: cluster of differentiation 33 (CD33), complement receptor 1 (CR1), membrane-spanning 4-subfamily A proteins (MS4AE and MS4A6S), bridging integrator 1 (BIN1), phosphatidylinositol-binding clathrin assembly protein (PICALM), CD2-associated protein (CD2AP), ephrin type A receptor 1 (EPHA1), apolipoprotein E (APOE), ATP-binding cassette transporter A7 (ABCA7) and clusterin (CLU). CD33, CR1, MS4AE, MS4A6S are involved in neuroinflammation which is considered to be a major contributing factor to AD if not a primary early underlying cause. The role of inflammation in AD has been reviewed [27]. BIN1, PICALM, CD2AP, EPHA1 are involved in various steps in endocytosis possibly related to the turnover of toxic Aβ and p-tau. The role of endocytosis in AD has been recently updated (Dominguez-Prieto et al, 2018). The risk CaMBPs involved in cholesterol metabolism are APOE, ABCA7 and CLU [26]. Certain APOE variants are high risk factors for the development of late onset AD [28]. A recent review of the function of these risk factor genes in microglia during Alzeimer's disease has added a new perspective to their importance in disease development [29],[30].

## 6. Calmodulin binds to critical receptors & ion channels

Metabotropic acetylcholine receptors (mAchR) are linked to AD because along with the loss of the neurotransmitter during AD, there is also a loss of receptors such as mAchR1 which is a proven CaMBP [31],[32]. In keeping with the central role of Ca^2+^ dyshomeostasis in AD, two Ca^2+^ channels, PMCA and NMDAR, bind to and are regulated by CaM. Aβ and CaM have a complex interplay in Ca^2+^ dysregulation. PCMA, the plasma membrane Ca^2+^-ATPase—a critical regulator of Ca^2+^-homeostasis—binds to and is activated by CaM and is the only brain Ca^2+^-pump that binds to and is inhibited by Aβ [33]. Upon CaM binding to PMCA, Aβ cannot bind to and inhibit PMCA allowing Ca^2+^ entry into cells already subjected to Ca^2+^-dysregulation. NMDA receptors (NMDAR, N-methyl-D-aspartate receptor) are heteromultimeric Ca^2+^ channels associated with memory and synaptic plasticity that bind Ca^2+^ via their NR1 subunits [34],[35]. The function of NMDAR in AD has been recently reviewed [36].

## 7. Conclusions

Calmodulin has been shown to have multiple proven or implied functions related to many of the key events and stages in the onset and progression of Alzheimer's disease. Both the formation of amyloid plaques and of neurofibrillary tangles each involve multiple CaMBPs. The turnover of plaques also is mediated by proteins shown to bind CaM. Central receptors and ion channels associated with the disease are CaMBPs. And, equally telling, is evidence that 11 early onset risk factor genes have motifs for CaM-binding including APOE, the protein most highly associated with risk for the disease. It seems there is enough evidence to argue that CaM should be moved to the top of the list of proteins to be studied in the goal to find effective therapeutics for dealing with both the onset and progression of the disease. To this end, inhibitors of the CaM-binding proteins CaMKII and CaN have been shown to reduce plaque burden, restore memory deficits and, even, reduce the incidence of dementia [37]–[39]. Popugaeva et al (2017) envision Ca^2+^ dyshomeostasis as a “therapeutic opportunity” [40]. We believe the data presented here suggests this opportunity is best focussed on taking the next step in Ca^2+^-signaling by targeting calmodulin and specific calmodulin-binding proteins.
